# A New Welfare Centre at Liverpool

**Published:** 1924-01

**Authors:** 


### A NEW WELFARE CENTRE AT LIVERPOOL.

A NEW Welfare Centre has been presented to Liverpool by the Carnegie United Kingdom Trust. The Centre is conveniently situated, lying between the Children's Infirmary and the site of the new Maternity Hospital, and is opposite the University School of Hygiene. The new institution, under the direction of the Public Health Department, will act as the headquarters of welfare work in the city, and should play an important part in the improvement of the general health of the community. Miss Haldane, Chairman of the Welfare Committees of the Carnegie United Kingdom Trust, in making the presentation, said that the aim of the Trust in taking up welfare work was that the Centres should serve as a model to the rest of the kingdom, and be concerned primarily with preventive work. The site at Liverpool was provided by the Corporation, and the whole of the £25,000 allocated by the Carnegie Trust was expended on the building and its equipment, which includes, besides consulting-rooms and lecture-rooms, two observation wards containing twelve beds, for the accommodation of sick children whom it may be thought desirable to keep under observation. The cost of maintenance will fall upon the Corporation, but a substantial grant will be received from the Ministry of Health. Work at the new Centre will begin in the New Year. While it was being opened at Liverpool another Carnegie Centre was being inaugurated at Shoreditch by Princess Mary.

